# Sulfamethoxazole-induced crystal nephropathy: characterization and prognosis in a case series

**DOI:** 10.1038/s41598-024-56322-9

**Published:** 2024-03-13

**Authors:** Ruben Azencot, Camille Saint-Jacques, Jean-Philippe Haymann, Vincent Frochot, Michel Daudon, Emmanuel Letavernier

**Affiliations:** 1 Physiology Unit, Service des Explorations Fonctionnelles Multidisciplinaires, AP-HP, Hôpital Tenon, 4 Rue de la Chine, 75020 Paris, France; 2 UMR S 1155, Sorbonne Université, 75020 Paris, France; 3 UMR S 1155, INSERM, 75020 Paris, France

**Keywords:** Sulfamethoxazole, Crystal, Crystalluria, Acute kidney injury, Biomarkers, Nephrology, Risk factors

## Abstract

Cotrimoxazole (Trimethoprim/Sulfamethoxazole-SMX) is frequently used in critically ill and immunocompromised patients. SMX is converted to *N*-acetyl-sulfamethoxazole (NASM) and excreted by the kidneys. NASM may form crystals in urine, especially in acid urine, that may induce a crystalline nephropathy. However, the imputability of crystals in acute kidney injury (AKI) has not been proven. We aimed to assess whether NASM crystals may promote AKI and to investigate risk factors associated with NASM crystalline nephropathy. Patients from Ile-de-France, France who developed AKI under SMX treatment introduced during hospitalization and had a crystalluria positive for NASM crystals were selected. Patients with excessive preanalytical delay for crystalluria or missing data regarding SMX treatment were excluded. We used the Naranjo score to assess the causal relationship between SMX and the development of AKI in patients with positive NASM crystalluria. Fourteen patients were included. SMX was the probable cause of AKI for 11 patients and a possible cause for 3 patients according to Naranjo score. Patients were exposed to high doses of SMX (but within recommended ranges), and most of them had a preexisting chronic kidney disease and were hypoalbuminemic. Urine pH was mildly acid (median 5.9). AKI occured more rapidly than expected after introduction of SMX (median 4 days) and recovered rapidly after drug discontinuation in most, but not all, cases. SMX is a probable cause of crystalline nephropathy. Monitoring of crystalluria in patients exposed to SMX may be of interest to prevent the development of crystalline nephropathy. Approval number of the study: BPD-2018-DIAG-008.

## Introduction

Since the 1980s, the use of Cotrimoxazole (Trimethoprim/Sulfamethoxazole), a combination of antimicrobials with broad-spectrum activity and proven efficacy, has been increasing, particularly in critically ill and immunocompromised patients^1–3^. Cotrimoxazole (CMX) is the drug of choice for the treatment of pulmonary pneumocystis.

Most of the metabolized sulfamethoxazole (SMX) is converted to N-acetyl-sulfamethoxazole (NASM) in the liver by N-acetylation. While NASM is mainly excreted by the kidneys via glomerular filtration and tubular secretion, it accounts for 60–65% of total SMX recovered in the urine^3–5^. Due to a higher incidence of toxicity in patients with acute kidney injury or pre-existing chronic kidney disease, dosage adjustment of CMX and close therapeutic monitoring are required, especially for patients with impaired glomerular filtration rate (GFR)^6,7^. A functional effect of Trimethoprim (TMP) on tubular creatinine secretion has been extensively characterized, which may artificially increase serum creatinine levels, even in the absence of acute kidney injury^8–11^.

In addition to its functional effect, it has been shown that CMX, and more specifically SMX, can cause acute kidney injury through either immunoallergic tubulointerstitial damages or acute tubular lesions due to drug precipitation^12,13^. These mechanisms have been described since the first use of sulfonamide antibiotics^14–18^.

Immunoallergic tubulointerstitial nephropathy is now recognized as a common mechanism of acute kidney injury secondary to SMX^19^.

Tubular lesions induced by intratubular precipitation of sulfonamides crystals have been described histologically since the 1940s^14,20^. Various types of sulfonamide crystals were detected in the urine, suggesting that they were responsible for the tubular kidney damage^21–24^.

Beginning in the 1970s, several clinical cases or case series of acute kidney injury associated with the presence of urinary crystals of NASM were published^25–27^. The advent of Fourier transform infrared (FTIR) spectroscopic analysis has allowed confirmation of the nature of the crystals and support for this mechanism^28–31^. The latest and largest case series to date by Castiglione et al. reports seven cases with NASM positive crystalluria^32^.

Based on our extensive database, we conducted a retrospective observational study, which represents the largest cohort of NASM crystalluria cases to date.

The aim of our study was to assess the causal relationship between SMX and the development of acute kidney injury in patients with positive NASM crystalluria (named in this article “SMX-induced crystal nephropathy”), using an internationally recognized drug causality assessment score^33^. Additionally, we investigated the potential risk factors associated with acute kidney injury secondary to SMX crystalluria, including the cumulative dose of SMX. We analyzed the course of kidney function using patient curves as illustration, and patient prognosis. Furthermore, we studied the characteristics and morphology of the crystals, supported by photographic evidence.

## Methods

### Study patients and design

More than 3300 urine samples are analyzed yearly at the renal physiology laboratory of Tenon Hospital, received from multiple centers in the Ile-de-France region. The crystals are analyzed by light microscopy examination. If the morphology of the crystals is not typical, or if there is history of medication use that may result in crystal formation, a FTIR method is used to identify the crystals^31^.

We conducted a retrospective, observational, descriptive study based on the collection of crystalluria analyses performed between November 2017 and January 2022.

### Selection criteria

All positive crystallurias for *N*-acetyl-sulfamethoxazole crystals (NASM). Thirty NASM-positive crystalluria were collected. One patient had 4 positive analyses and 2 patients had 2 positive analyses. For patients who had multiple positive NASM crystalluria, we retained the data from the first one observed.

#### Inclusion criteria

1. CMX treatment introduced during hospitalization.
2. Acute kidney injury (AKI, according to K-DIGO 2012 definition) under CMX treatment.

#### Exclusion criteria

1. Excessive preanalytical delay for crystalluria: we considered only crystalluria analyzed the same day than urine collection and discarded therefore all analyses with a pre-analytical delay superior to 7 h.
2. Missing data regarding the administered doses of CMX.

This study was conducted in accordance with declaration No. 004 of the CNIL (National Commission for Data Protection and Liberties). It was declared to the data protection office and registered in the general registry of treatment of the AP-HP (Assistance Publique-Hôpitaux de Paris) (approval number BPD-2018-DIAG-008).

### Data collection

Data collection was performed in an Excel spreadsheet using crystalluria number used in the physiology department. Anonymized data were retrieved from the patient’s hospitalization records using the Orbis computerized software, or from paper records of the patients in the absence of Orbis software.

The collected data included the following: patient background (age, sex, body mass index [BMI], medical history of hypertension, diabetes, ischemic heart disease, chronic obstructive pulmonary disease [COPD], solid cancers of hematological malignancies, immunosuppression including long-term corticosteroid therapy, chronic kidney disease [CKD]), usual treatment, baseline kidney function (creatinine and eGFR according to CKD-EPI 3–8 months before hospitalization), main diagnosis, clinical data at admission (temperature, hemodynamic and respiratory parameters, IGS-2 score), in-hospital clinical data (indication for CMX, dosages and duration of CMX treatment, administration of other potentially nephrotoxic drugs such as non-steroidal anti-inflammatory drugs [NSAIDs], angiotensin-converting enzyme [ACE] inhibitors, angiotensin receptor blockers [ARBs], β-lactams, glycopeptides, aminoglycosides, quinolones, anti-viral drugs, and calcineurin inhibitors), laboratory data at admission (serum creatinine, eGFR, serum urea, serum sodium, serum potassium, blood cells count and platelet count, liver function tests, CRP, and serum albumin) and during stay (blood electrolytes, urinary electrolytes, proteinuria on sample, cytological examination of urine).

Follow-up data included: survival or death at last follow-up (April 2022), kidney function assessment (creatinine and eGFR using CKD-EPI equation) 3–6 months after hospitalization.

### Study objectives

The primary objective of the study was to determine the causality of CMX in Acute Kidney Injury (AKI) using the Naranjo International Scale^33^. The Naranjo causality score was independently assessed by two clinical nephrologists who were investigators in the study. In case of discordance between the two evaluators, the lower Naranjo score was considered.

Kidney function trend curves, considering the exact doses and timing of SMX administration, along with the consideration of confounding factors such as hemodynamic stability, co-administration of potentially nephrotoxic drugs or vasopressors, and corticosteroid therapy, allowed the most optimal assessment of the score.

In a second step, patients with a “probable” causality (Naranjo score ≥ 5) or "certain" causality (Naranjo score > 9) of SMX-induced crystal nephropathy, as determined by both investigators, were selected.

The secondary objectives of this study were to evaluate various aspects in patients with SMX-induced crystal nephropathy. First, we aimed to identify the risk factors for AKI associated with this condition, including the daily dosage and cumulative dose of SMX before crystalluria occurred. Second, we sought to examine the trends in kidney function over time by analyzing plasma creatinine and urea. We also characterized the NASM urinary crystals in terms of size, number, and assessed urine pH and urine density. Additionally, we aimed to determine the profile of AKI secondary to SMX-induced crystalluria by assessing daily diuresis, urinary ionogram, proteinuria, urinary sediment, severity of kidney injury based on the K-DIGO classification, presence of hyperkalemia, and co-administration of other nephrotoxic drugs or vasopressor drugs. Furthermore, we aimed to investigate the prognosis of AKI secondary to SMX-induced crystal nephropathy by assessing mortality, kidney function (creatinine, CKD-EPI) at 3–6 months after positive crystalluria, kidney function at the last follow-up, and survival at the last follow-up.

### Data analysis

Median values and interquartile ranges (IQR) are presented for quantitative variables, and percentages for qualitative variables. These calculations were performed using Microsoft Excel software.

### Ethical approval and consent to participate

This study was conducted in accordance with declaration No. 004 of the CNIL (National Commission for Data Protection and Liberties). It was declared to the data protection office and registered in the general registry of treatment of the AP-HP (Assistance Publique-Hôpitaux de Paris), and received the approval number: BPD-2018-DIAG-008.

## Results

### Study patients

**Figure 1:**
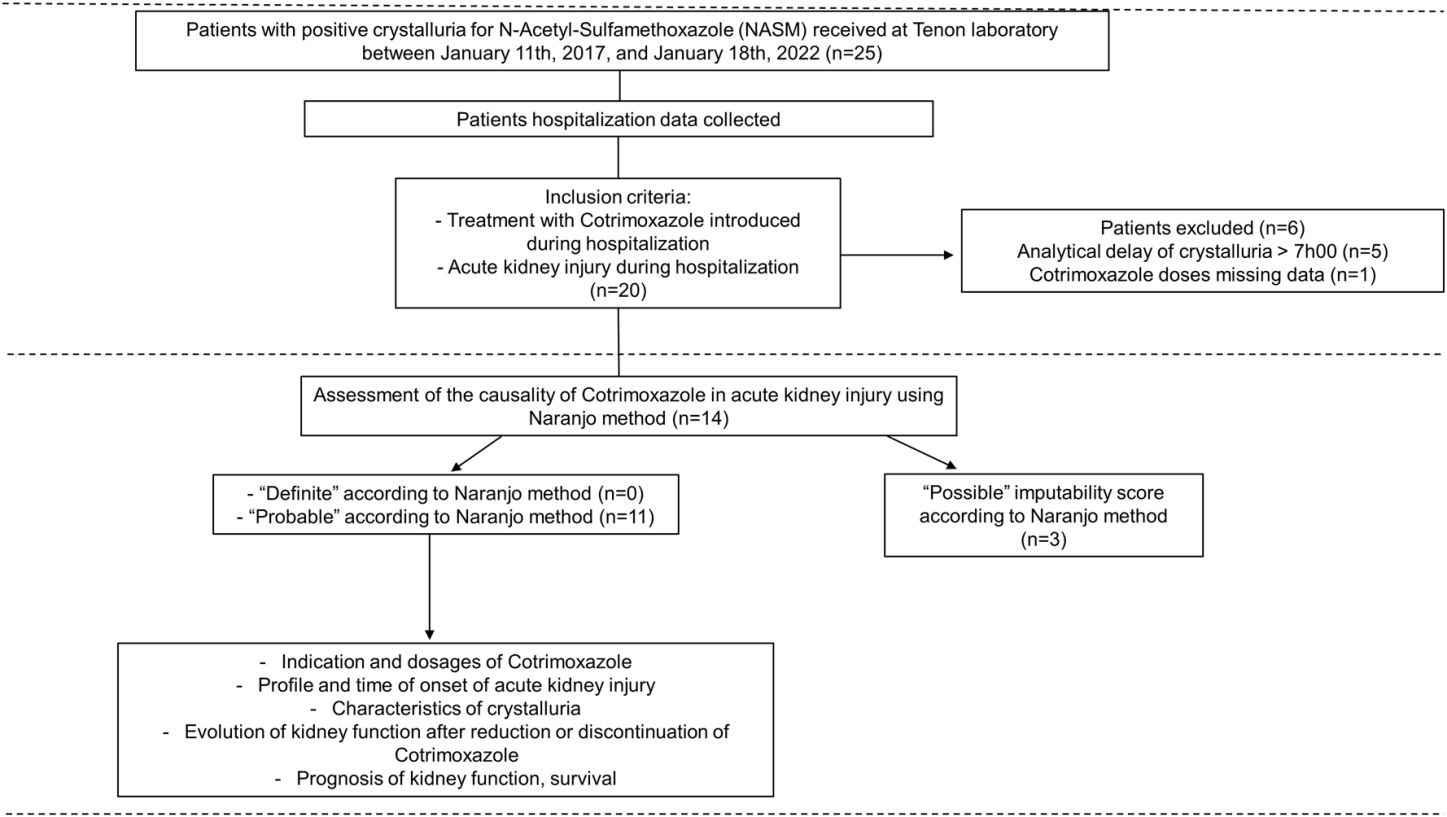
Flowchart.

The flowchart details patients selection criteria (Fig. 1). A total of 25 patients with NASM-positive crystalluria were identified at Tenon Hospital laboratory between November 2017 and January 2022.

Five patients did not meet the inclusion criteria for the following reasons: three patients did not have AKI, and two patients were receiving long-term CMX prior to hospitalization either at prophylactic or therapeutic doses (Supplemental Table 1).

Six patients were excluded: Five because the preanalytical time before crystalluria was ≥ 7 h, and one because data on administered doses of CMX were missing.

Fourteen patients were included. After evaluation by the Naranjo method, 3 patients had “possible” imputability of CMX to acute kidney injury and 11 patients had “probable” imputability (Supplemental Table 2). Regarding the 3 patients with “possible” imputability, two received other potential nephrotoxic drugs (aminoglycosides) and one had a delayed degradation of kidney function after introduction of SMX. Regarding the 11 patients with probable imputability, no other cause for kidney injury was evidenced, kidney function decreased rapidly after SMX treatment and improved after drug discontinuation.

### Patient characteristics

Demographic, medical history, clinical, and laboratory data at admission of the 11 patients are summarized in Table 1.

**Table 1:** Patient characteristics, clinical, and laboratory data at admission.

Total number of patients	11			
	NA			
Age (years)	0	72 [56.5–77]		
Male gender	0	7 (63.63%)		
Body mass index (kg/m^2^)	0	27.9 [25.8–31.22]		
Arterial hypertension	0	7 (63.64%)		
Mellitus diabetes	0	2 (18.18%)		
Ischemic cardiopathy	0	0 (0.00%)		
COPD	0	4 (36.36%)		
Immunocompromised	0	11 (100.00%)	Corticosteroid therapy	9 (81.82%)
			Chemotherapy	1 (9.09%)
			HIV infection	1 (9.09%)
History of solid cancer	0	8 (72.72%)		
Chronic kidney failure	0	7 (63.64%)	Stage 3A Moderate (GFR = 45–59 mL/min)	1 (9.09%)
			Stage 3B Moderate (GFR = 30–44 mL/min)	2 (18.18%)
			Stage 4 Severe (GFR = 15–29 mL/min)	4 (36.36%)
Glomerular filtration rate (GFR) 3–8 months before admission (mL/min/1.73m2)	3	32 [22.5–42]		
Clinical data at admission				
IGS-2 score at admission	2	37 [33-48]		
Fever	0	0 (0.0%)		
Septic shock /Use of Noradrenalin at admission	0	0 (0.0%)		
Use of Noradrenalin at admission	0	0 (0.0%)		
Biological data at admission				
Serum creatinine (µmol/L)	0	140 [105.5–183]		
Glomerular filtration rate (GFR) (mL/min/1.73m2)	0	44.5 [22.35–54]		
Blood urea (mmol/L)	0	11.4 [9.35–15.8]		
Serum albumine (mmol/L) (within 4 days after admission)	5	29.4 [28.2–30.72]		
Aspartate transaminase (AST) (U/L)	0	22 [21–39.5]		
Alanine transaminase (ALT) (U/L)	0	19 [14.5–35]		
Protein C reactive (mg/mL)	2	92.5 [44–135.3]		
Platelets (G/L)	0	205 [140–305]		
White blood cells (G/L)	0	8.69 [0.55–24.94]		
Neutrophils (G/L)	2	7.13 [6.39–7.87]		
Lymphocytes (G/L)	2	0.47 [0.4–0.59]		

Categorical variables are presented as count (%).

Continuous variables are presented as median [interquartile].

*NA* data not available.

Seven of the 11 patients (63.63%) were males. The median age of the patients was 72 years. Seven patients (63.63%) had hypertension, and two patients (18.18%) had diabetes. Eight patients (72.72%) had a history of solid cancer.

All patients were immunocompromised, with 9 of them on long-term corticosteroid therapy (81.82%), one receiving chemotherapy for solid cancer, and one being HIV-positive.

The median estimated glomerular filtration rate (eGFR) according to CKD-EPI (in mL/min/1.73m2) 3–8 months prior to admission was 32 mL/min/1.73m2. The majority of patients (63.64%) had chronic kidney disease (CKD): four stage 4 (36.36%), two stage 3B (18.18%), and one stage 3A (9.09%).

The median plasma creatinine level at admission was 140 μmol/L.

All patients for whom albumin was measured were hypoalbuminemic, with a median albumin level of 30.485 g/L.

As mentioned, nine patients were already receiving oral corticosteroids for various indications when CMX was introduced. These indications included intracranial hypertension due to brain metastases (200 mg daily), cauda equina syndrome, systemic disease (5 mg daily), cordarone-induced pneumonitis (5 mg daily) and recent lung transplant rejection, previously treated with boluses a month earlier.

Of these 9 patients, 4 underwent corticosteroid dose escalation during hospitalization, receiving bolus doses (ranging from 120 to 500 mg per day intravenously), followed by maintenance doses of 1 mg/kg per day (oral or intravenous, depending on the patient). The main indication for this escalation was suspected pneumocystis.

One patient had not previously received a maintenance dose and was started on intravenous corticosteroids at 40 mg daily due to suspected pulmonary pneumocystis.

### Data on CMX, and other nephrotoxic medications

SMX doses data prior to crystalluria are shown in Table 2.

**Table 2:** Doses of SMX, and median delays after SMX introduction.

	NA	Route of administration	Dose (n = 11)
Initial dosage, cumulative doses of SMX prior to crystalluria			
Initial dose of SMX	0	Intravenous (mg/kg/day) 11/11	55.81 [39.25–66.78]
Cumulative SMX dose received prior crystalluria	0	Intravenous (mg) 8/11	17,800 [16800–29700]
	0	Intravenous + oral (mg) 3/11	70,400 [53600–86400]

	NA	Number of days	
Median delays after SMX introduction (first and third quartiles)			
Initiation of SMX – Acute kidney injury	0	4 [3.5–5]	
Initiation of SMX – Crystalluria	0	6 [5–13]	
Initiation of SMX – Peak plasma creatinine	0	7 [6–12.5]	

Data are expressed as median (interquartile range).

*NA* data not available.

Initially, all patients received SMX intravenously, followed by oral administration in 3 patients before crystalluria assessment. The median daily dosage at introduction of SMX was 55.81 mg/kg/day (IV). The median cumulative dose for patients (8 out of 11) who received SMX only intravenously was 17,800 mg. The median cumulative dose for patients (3 out of 11) who received SMX intravenously and then orally was 70,400 mg, regardless of the route of administration.

Median time intervals (first and third quartiles) between SMX introduction and occurrence of crystalluria, acute kidney injury, and peak plasma creatinine levels (in µmol/L) are depicted in Table 2.

**Figure 2:**
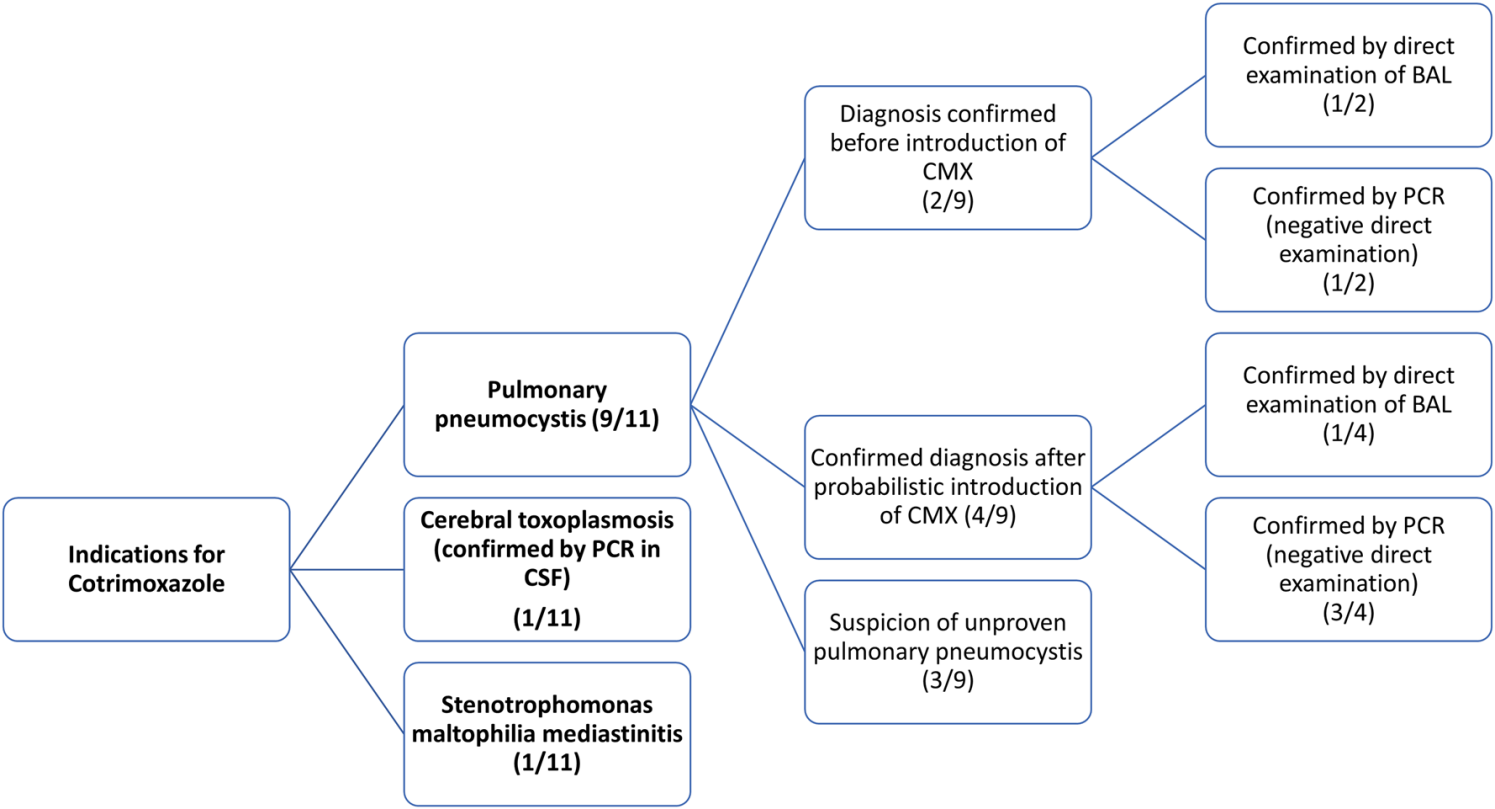
Distribution of CMX indications among patients (CMX: CMX, CSF: cerebrospinal fluid, BAL: bronchoalveolar liquid).

The indications for CMX use are presented in Fig. 2.

Nine patients received CMX for the treatment of pulmonary pneumocystosis, 2 had a confirmed diagnosis at the time of initiation, 4 patients had a secondary diagnostic confirmation after initiation, and 3 were suspected but did not have microbiological confirmation.

One patient received CMX for the treatment of confirmed cerebral toxoplasmosis by PCR in cerebrospinal fluid, and one for *Stenotrophomonas maltophilia* mediastinitis.

### Data on changes in kidney function on CMX based on plasma creatinine and urea

Data regarding changes in serum creatinine and urea levels are presented in Table 3.

**Table 3:** Changes in serum creatinine and urea levels (n = 11).

Serum creatinine (µmol/L)	
Baseline (admission day)	140 [105.5–183]
Day of crystalluria	289 [187.5–365.5]
Serum urea (mmol/L)	
Baseline (admission day)	11.4 [9.35–15.8]
Day of crystalluria	26.00 [18.25–29.45]

Data are expressed as median (interquartile range).

**Figure 3:**
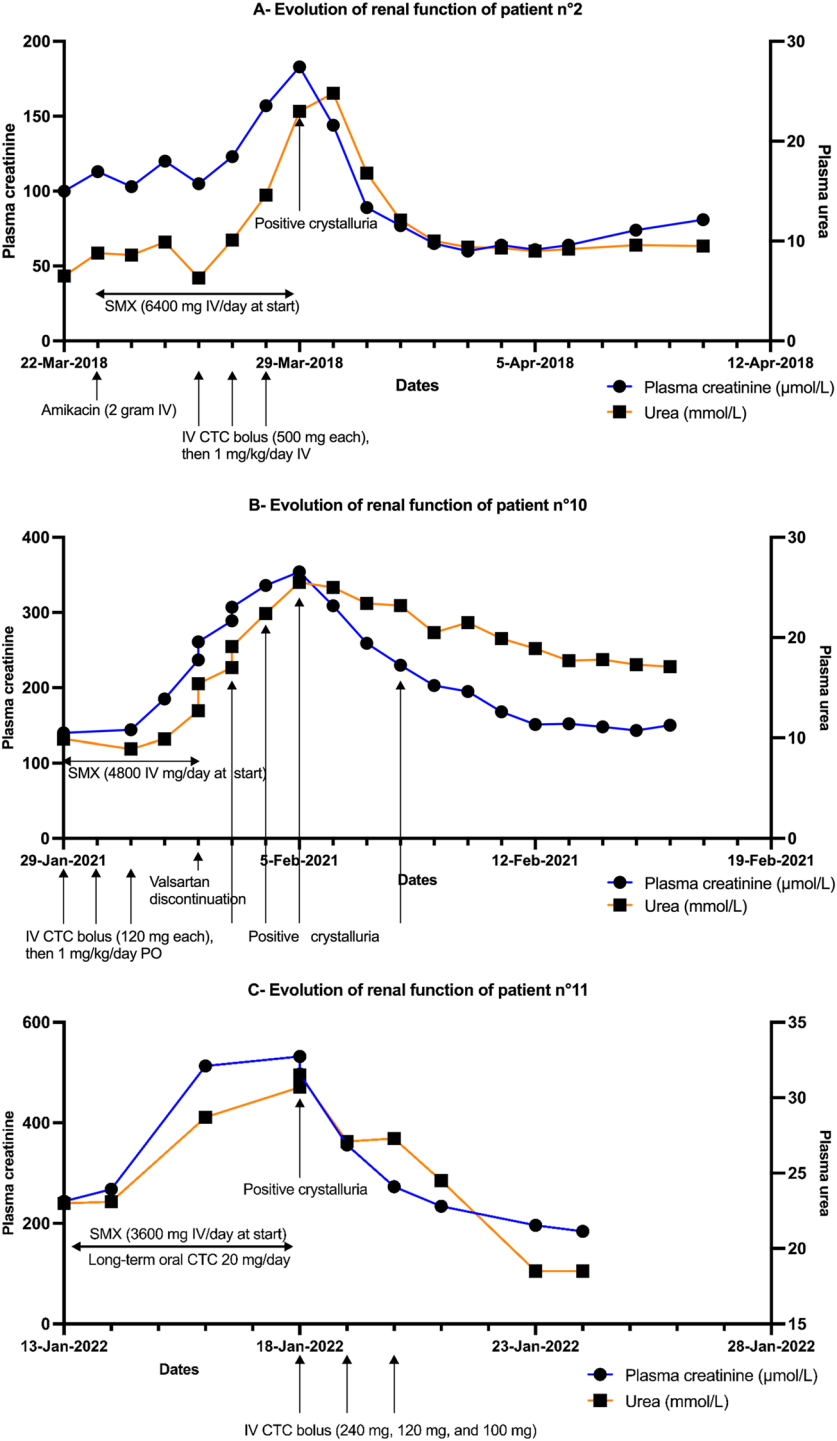
(**A**–**C**) Evolution of kidney function of patients n°2, n°10, and n°11 after introduction of SMX. *All drugs with a potential impact on kidney function have been added to the graph.* (SMX: Sulfamethoxazole ; CTC : Corticosteroid therapy ; IV: Intravenous ; Jan : January ; Mar : March ; Apr : April ; Feb: February).

Representative evolution of serum creatinine and urea in individuals after the introduction of SMX during hospitalization is depicted in Fig. 3A–C. Patient N°10 (Fig. 3B) is one of the two patients in our study for whom several crystalluria tests were performed.

### Acute kidney injury profile, use of nephrotoxic drugs, complications during hospitalization data (Tables 4 and 5)

**Table 4:** Acute kidney injury profile of patients (n = 11).

	NA	
Severity (KDIGO classification)	0	
K-DIGO 1		1 (9.09%)
K-DIGO 2		5 (45.45%)
K-DIGO 3		5 (45.45%)
Hemodialysis required	0	1 (9.09%)
Diuresis on the day of crystalluria (mL/day)	2	800 [600–2100]
Serum creatinine on the day of crystalluria (µmol/L)	0	289 [187.5–365.5]
Creatinine peak during the stay (µmol/L)	0	310 [193.5–369.5]
Blood urea on the day of crystalluria (mmol/L)	0	26.00 [18.25–29.45]
Urinary sodium/potassium ratio on the day of crystalluria	0	2.92 [0.74–7.88)
Urinary creatinine on the day of crystalluria (mmol/L)	1	5.41 [4.275–7.075]
Urinary urea on the day of crystalluria (mmol/L)	1	135.9 [114.975–230.25]
Urinary/plasma urea ratio on the day of crystalluria	1	6.43 [4.82–9.09]
Serum potassium level on the day of crystalluria (mmol/L)	0	4.9 [3.85–5.35]
Occurrence of hyperkalemia (> 5 mmol/L)	0	4 (36.36%)
Significant proteinuria (> 30 mg/mmol)	3	6 (54.54%)
Significant leukocyturia (> 10/mm^3^)	1	5 (45.45%)
Significant hematuria (> 10/mm^3^)	1	6 (54.54%)

Categorical variables are presented as count (%).

Continuous variables are presented as median [interquartile].

*NA* data not available.

**Table 5:** Use of other nephrotoxic drugs and complications during hospitalization (n = 11).

	NA		
Initiation or continuation of other potentially nephrotoxic drugs at admission	0	8 (72.72%)	
		Aciclovir	2 (18.18%)
		Amikacin	2 (18.18%)
		Gentamicin	1 (9.09%)
		Calcineurin inhibitors	1 (9.09%)
		Amoxicillin	1 (9.09%)
		Ciprofloxacin	1 (9.09%)
		Amphotericin B	1 (9.09%)
		Piperacillin/tazobactam	3 (27.27%)
		ACEi/ARB	1 (9.09%)
		NSAIDs	0 (00.00%)
Complications during hospital stay			
Septic shock	0	0 (00.00%)	
Use of Noradrenalin	0	1 (9.09%)	
Acute respiratory failure requiring orotracheal intubation	0	4 (36.36%)	
Fever > 7 days	0	0 (00.00%)	

Categorical variables are presented as count (%).

*NA* data not available, *ACEi* angiotensin-converting enzyme, *ARB* angiotensin receptor blockers, *NSAIDs* non-steroidal anti-inflammatory drugs.

Acute kidney injury profile data are presented in Table 4, including severity, daily diuresis, urine ionogram, proteinuria, urine sediment, and serum potassium levels.

Use of other nephrotoxic medications, and complications during hospitalization data are presented in Table 5.

### Kidney and patient outcomes (Table 6)

**Table 6:** Long-term Kidney and Survival outcomes in study patients (n = 11).

	NA	
Kidney outcomes		
Serum creatinine 3–6 months after crystalluria (µmol/L)	1	126.5 [117.25–138.25]
Glomerular filtration rate (GFR) 3–6 months after crystalluria (mL/min/1.73m2) (CKD-EPI)	1	39 [33.575–56.35]
Serum creatinine at last follow-up (µmol/L)	2	152 [124–166]
Glomerular filtration rate (GFR) at last follow-up (mL/min/1.73m2) (CKD-EPI)	2	38 [32–48.4]
End-stage renal disease (ESRD)	2	1 (9.09%)
Survival outcomes		
Death during hospitalization	0	1 (9.09%)
Death at last follow-up	0	4 (36.36%)

*NA* data not available.

A patient with pre-existing stage 4 chronic kidney disease has not recovered kidney function and has progressed to end-stage renal disease following hospitalization.

Two patients died between the time of the crystalluria and the last follow-up. One patient died during hospitalization due to acute respiratory distress syndrome (ARDS), while the other died 17 months after discharge due to respiratory failure secondary to interstitial pneumonia caused by Amiodarone use.

### Characteristics of NASM positive crystalluria: size, number, urine pH, urine density (Table 7)

**Table 7:** Characteristics of NASM positive crystalluria (n = 11).

	NA	
Urine volume (mL)	0	70 [15–200]
Urine osmolality (mOsm/kg)	7	445.5 [318–575.5]
Urine pH (dipstick)	0	5.9 [5.3–6.05]
Urine gravity (dipstick)	0	1025 [1017.5–1030]
Number of NASM crystals (/mm^3^)	3	79.5 [32.5–265]
Maximum size of NASM crystals (µm)	0	23 [8.5–26.5]
Average size of NASM crystals (µm)	0	10 [4.75–14]

*NA* data not available.

The median urine pH was 5.9. The median urine specific gravity was 1025. The median number of NASM crystals was 79.5/mm^3^.

**Figure 4:**
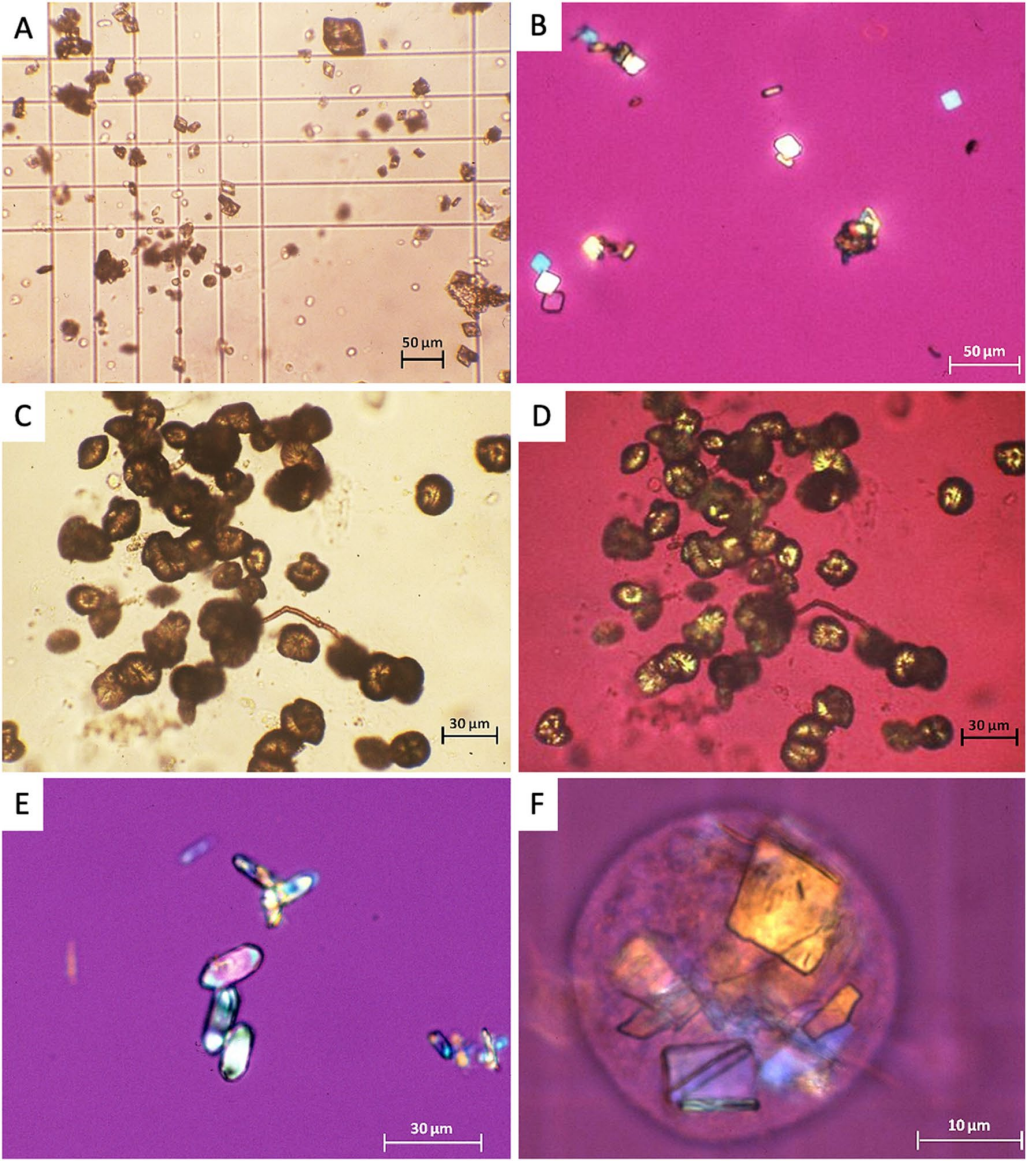
*N*-Acetyl-Sulfamethoxazole crystals Representative NASM crystals from patients urine. (**A**) and (**B**): lozenges and parallelepipeds in bright field (**A**) and polarized light (**B**); (**C**) and (**D**): spheroids, or “shocks of wheat” shapes in bright field (**C**) and polarized light (**D**); (**E**) ovals, crystals mimicking calcium oxalate monohydrate; (**F**) crystals phagocytosed in a cell that could be a macrophage.

Representative NASM urinary crystals are shown in Fig. 4.

## Discussion

To our knowledge, this study is the largest case series of SMX-induced crystalline nephropathies to date. This comprehensive investigation involved the collection of clinical data associated with SMX-induced crystalluria, enabling us to identify risk factors and characterize kidney outcomes associated with SMX-induced crystalline nephropathy. Our aim was also to study the kinetics of impaired kidney function upon SMX introduction and after drug discontinuation, to assess the prognosis and kidney survival of affected patients, and to analyze the characteristics of crystalluria (including crystal morphology, urinary pH and density).

Previous studies have examined the population and risk factors for acute kidney injury on CMX^12,13,34^.

The cohort consisted mainly of elderly patients, frequently hypertensive, which aligns with findings from previous studies^12,13,34^. All patients in our study were immunocompromised, a condition leading to confirmed or suspected opportunistic infections that required SMX therapy. Of the immunocompromised patients, 9 of 11 were on long-term corticosteroid therapy, and only one was affected by HIV which is a notable finding in comparison with other studies including more HIV-positive patients.

The majority of patients in our study had preexisting chronic kidney disease (63.6%), including 4 patients with stage 4 CKD. Previous studies suggested that preexisting chronic kidney disease is a risk factor for SMX-induced acute kidney injury^12,35^.

Based on Le Gall et al., a median IGS-2 score of 37 corresponds to an expected hospital mortality rate of approximately 21.7%, a severe condition but an intermediate risk for patients admitted to intensive care units^36^.

Four patients exhibited hyperkalemia, which was secondary to acute kidney injury. Trimethoprim, which has an inhibitory effect on tubular potassium secretion, might have contributed to this condition^37^.

The FDA (Food Drug Administration) recommends a daily dose of 800 mg for the treatment of common infections in adults, while the recommended dose for pneumocystis pneumonia is 75–100 mg/kg/day of SMX, with dose adjustments based on kidney function.

In our study, all patients were initially treated with intravenous SMX, with a median daily dose of 55.81 mg/kg/day at introduction. The risk of AKI associated with CMX use has been studied, but the literature has reported inconsistent findings regarding the relationship between dosage and AKI risk.

Fraser’s and Shimizu’s studies provided conflicting results regarding a dose-dependent relationship between SMX and AKI^12,13^. Fraser et al. did not find a significant relationship whereas Shimizu et al. identified a dose-dependent effect^12^.

In our study, the median time to onset of AKI after SMX introduction was 4 days (IQR 3.5–5 days), shorter than in Fraser’s study (6.5 days) and Shimizu’s study (8 days [IQR, 5–16 days])^12,13^.

However, these studies did not distinguish between the different mechanisms of acute kidney injury secondary to CMX use. They probably included patients with SMX-induced crystalline nephropathy, tubulo-interstitial nephropathy due to an immuno-allergic mechanism and, possibly, functional increases in plasma creatinine. Leucocytes were identified in the urine of 5 patients with probable crystalline nephropathy. No eosinophilic cells count was performed. We cannot rule out the participation of immuno-allergic processes but the rapid improvement of kidney function after SMX withdrawal does not support this hypothesis.

Our study focused specifically on SMX-induced crystalline nephropathy, necessitating consideration of this distinct parameter in comparisons with previous studies. According to our observations, risk factors for SMX-related AKI previously identified in former studies are actually risk factors for SMX-related crystalline nephropathy.

We observed NASM crystals in 5 patients who were not included for two reasons (Supplemental Table 1). First, two patients developed AKI but were treated with long-term CMX (Supplemental Table 1).

Second, three patients did not develop AKI: one was treated with short-term oral CMX for an exacerbation of COPD, another received long term prophylaxis for pneumocystosis, and the last patient was treated for suspected pneumocystis intravenously (Supplemental Table 1).

NASM crystals may therefore be observed in urine in the absence of clinically evident kidney lesions. Further prospective studies are required to characterize whether NASM-positive crystalluria is an independent risk factor for AKI, as recently evidenced for amoxicillin^38,39^. One may hypothesize that intermittent and sparse crystals may be less toxic for kidney tubule than a huge number of crystals: the number of crystals in urine and the global crystalline volume should be assessed in future studies.

Overall, our findings suggest the importance of very early monitoring of kidney function in patients receiving high doses of SMX for pulmonary pneumocystosis, and the need for further prospective studies evaluating the predictive value of crystalluria in patients exposed to SMX.

A study by Berglund et al. in 1975 showed that TMP caused a significant increase in plasma creatinine levels without affecting GFR^10^. Lee observed a similar effect studying the influence of TMP on tubular transport of organic anions and cations in rat kidney tissue^11^. Shouval noted that CMX caused transient changes in plasma creatinine and creatinine clearance^8^. In 1981, Kainer found no significant changes in GFR measured by EDTA clearance in healthy adults treated with CMX^9^.

In our study, we demonstrate that patients with SMX-induced crystalline nephropathy exhibit acute kidney injury, confirmed by the comparable evolutionary profiles of urea and plasma creatinine levels. The inhibitory effect of TMP on tubular creatinine secretion exists and is confirmed by the dissociation between creatinine and urea levels observed with CMX, as shown in individuals in Fig. 3A–C, but the simultaneous increase in serum creatinine and urea confirms the onset of AKI. Of note, the increase in urea cannot be attributed to the use of corticosteroids, as it coincides with the introduction of CMX and decreases significantly after the drug is discontinued. The evolution of kidney function in Fig. 3A–C illustrates this trend.

In the Shimizu study, univariate logistic regression analysis revealed that concomitant use of loop diuretics, β-lactams, glycopeptides, and aminoglycosides were independent variables associated with the development of acute kidney injury (AKI) under CMX therapy^13^.

Our aim was to rule out the involvement of other factors in the development of AKI, such as other nephrotoxic agents (including aminoglycosides, vancomycin….) or hemodynamic alterations. Comprehensive data collection, facilitated by the fact that patients were hospitalized in intensive care units, enabled in-depth analysis of each case, including drug dosages, administration timing and other relevant clinical parameters.

The Naranjo score was used to assess the imputability of SMX crystals in cases of AKI, while ruling out other possible causes. Key factors that contribute significant points to Naranjo’s causality assessment score include the time interval between the introduction of the drug and the onset of the adverse reaction, and the absence of other potential causes or other drugs responsible for the adverse reaction^33^. The presence of other nephrotoxic factors, including drugs, was therefore taken into account, and the SMX was considered as the probable cause (score ≥ 5) of AKI for 11 patients. In most of patients, no other potential cause of AKI was identified. Some patients received other potential nephrotoxic drugs but the timescale analysis and the evolution of kidney function did not support the role of these treatments in the onset of AKI. Only one patient received vasopressor amines, in low doses and briefly.

The kidney prognosis of the patients in our study was generally favorable, as only one patient developed end-stage renal disease, and this patient was already in stage 4 chronic kidney disease prior to the hospitalization. Obviously, the identification of crystals in urine led to SMX withdrawal and may have modified the evolution of kidney function.

The use of sulfonamides has been linked to the presence of crystals in urinary sediments since decades^21,23–25^. The study of crystal structures has been based primarily on individual clinical cases and it was shown that crystal morphology varies with the type of sulfonamide, and sometimes for a same type of sulfonamide^21,40^.

Prien and Frondell already identified various types of crystals in the urine sediments of patients receiving sulfonamides in 1940^21^. Different shapes were found such as arrowheads, thick convex lenses, rosettes, hexagons, millstones, sheaves (or shocks) of wheat, or dumbbells. The appearance of sulfonamide crystals in the urine is closely related to the solubility of their compounds and acetylated derivatives^21,23^.

Daudon et al. conducted a retrospective study to determine the frequency and nature of spontaneous crystalluria in a population without uronephrologic pathology^41^. They observed 466 positive crystalluria (7.6%) out of 6100 urine samples analyzed. Drug crystals accounted for 8.5% of all positive crystalluria. NASM crystals were largely predominant, found in 27 of 466 samples (5.8%), mostly in pure form (4.9%, vs. 0.9% mixed forms), and equally distributed between the sexes. The mean urinary pH of the urine samples was 5.7. They were rarely associated with urinary stones (0.1% of cases).

Few other cases of NASM crystalluria have been reported in the literature^25–27,30,42–46^. The largest retrospective case series reported on NASM-positive crystalluria to date was published in 2018 by Castiglione et al.^42^. It included seven patients with various bacterial infections. FTIR spectrophotometric analysis authenticated the NASM crystals, as in the studies of Daudon et al.^30,31,41^. Lower doses of SMX were associated with rectangular-shaped crystals, whereas higher doses resulted in larger, irregularly shaped crystals^32^.

We found no correlation between the dosage of SMX and crystal morphology. However, we frequently observed various crystal shapes present at the same time in an individual.

The majority of crystal morphologies align with those documented in previous cases, including lozenges, hexagons, flower-like, globules or spheroids, mushrooms or dumb-bells, shocks or sheaves of wheat (which appear to describe a similar morphology using different terms)^27,30,32^. Interestingly, we also observed in some cases intracellular crystals, probably in macrophages, suggesting that NASM crystals may induce an inflammatory response.

The main potential risk factors identified for SMX-induced crystal nephropathy are acidic urine pH, hypovolemia resulting in low diuresis, hypoalbuminemia, high urine concentrations of SMX and NASM, and underlying chronic kidney disease^26,30,32^.

Only half of the patients had low urinary pH levels, with a median value of 5.9. This observation is in line with some cases reported by Castiglione^32^. Hence, ruling out SMX-induced crystalline nephropathy solely based on non-acidic urine criteria may not be justified.

We observed consistently high urinary gravity exceeding 1015 for all patients (median 1025), indicating concentrated urine and reduced urine output.

Hypoalbuminemia may increase the risk of crystalluria by increasing plasma and free urinary concentrations of NASM^5,26,30,47^. In our study, all patients with available albumin levels (missing data for 5 patients out of 11) were hypoalbuminemic.

Furthermore, it is noteworthy that patients in our cohort received notably high initial doses of SMX, all administered intravenously. For three patients, a transition to oral administration was initiated as kidney function began to decline.

Patients with pre-existing chronic kidney disease are also at high risk, as confirmed by our observations^27,30,42^.

In summary, our findings highlight the importance of low diuresis volume, hypoalbuminemia, and notably, high doses of SMX, alongside pre-existing chronic kidney disease, as key indicators for potential SMX-induced crystalline nephropathy risk. By contrast, low urine pH does not seem mandatory to develop AKI in this context. Clinicians should pay special attention to these factors when initiating CMX therapy.

Crystalluria, although not commonly conducted in all centers, holds potential as a diagnostic tool for identifying SMX-induced crystalline nephropathy. The wide range of morphologies exhibited by NASM crystals, which can occasionally resemble other crystals like uric acid, cystine, or calcium oxalate monohydrate, emphasizes the necessity of an experienced biologist and the utilization of FTIR spectrophotometry analysis to definitively ascertain their composition^30^.

Our study has several limitations. First, it is important to acknowledge that our study design is retrospective, which poses inherent limitations. A prospective study, similar to those conducted for Amoxicillin, would provide more robust evidence^38,39^. This type of study would involve systematically conducting crystalluria assessments on all hospitalized patients receiving therapeutic doses of SMX, allowing for the observation of positive crystalluria and the early characterization of patients who develop acute kidney injury. By incorporating a prospective approach, we could gather more reliable data on the incidence and progression of SMX-induced crystalline nephropathy.

In addition to the retrospective nature of our study, another limitation is the small sample size, which restricts our ability to perform comprehensive data analyses to confirm the risk factors associated with SMX-induced crystalline nephropathy.

Intriguingly, despite the Naranjo score indicating probable crystalline nephropathy, there are no known reports of intratubular NASM crystals in kidney biopsies from patients with acute kidney injury. This might be attributed to biopsy preparation methods. Recently, the presence of Amoxicillin crystals in kidney biopsies has been demonstrated, even though the link between Amoxicillin crystals and acute kidney injury was already established^48^. Amoxicillin crystals tend to disappear during paraffin embedding but can be identified in frozen sections. A similar hypothesis could apply to NASM crystals, suggesting that frozen sections in patients with a high likelihood of SMX-induced crystalline nephropathy would be worthwhile to investigate. Moreover, most of patients with presumed SMX-induced crystalline nephropathy do not usually undergo kidney biopsies due to the mostly favorable outcomes upon discontinuation of the medication, this was the case for all patients included in this study.

Despite these limitations, our study provides valuable clinical information that will be of help to design future prospective studies. SMX is a widely used drug and caution should be exercised when administering high doses, particularly in patients with chronic kidney disease (CKD), as they appear to be at increased risk of SMX-induced crystalline nephropathy. Close monitoring of kidney function and dosage adjustments are essential in these cases. Thus, in the context of positive crystalluria, dose reduction of SMX is probably appropriate, particularly if crystalline nephropathy is suspected in patients receiving high doses of CMX. Readjustment of the patient’s fluid balance by appropriate hydration, possibly alkaline if urinary pH is acidic, would in fact improve crystal solubility and minimize intratubular precipitation^24,29^. If crystalline nephropathy is strongly suspected, switching to pentamidine would be an option.

These observations emphasize the significance of utilizing early crystalluria as an important warning sign in the management of these patients, and to develop a prospective evaluation of crystalluria value in patients exposed to high doses of SMX.

### Supplementary Information

Supplementary Table 1.

Supplementary Table 2.

## Data Availability

The datasets used and/or analyzed during the current study are available from the corresponding author on reasonable request.

## Abbreviations

AP-HP: Assistance Publique-Hôpitaux de Paris
AKI: Acute kidney injury
BAL: Bronchoalveolar liquid
CMX: Cotrimoxazole
CNIL: National Commission for Data Protection and Liberties
CKD: Chronic kidney disease
CSF: Cerebrospinal fluid
CTC: Corticosteroid therapy
FTIR: Fourier transform infrared
HIV: Human immunodeficiency virus
IV: Intravenous
NASM: *N*-acetyl-sulfamethoxazole
SMX: Sulfamethoxazole
TMP: Trimethoprim
