# Supplementary material for: Sulfamethoxazole-induced crystal nephropathy: characterization and prognosis in a case series
Source: Sci Rep. 2024 Mar 13;14:6078. doi: 10.1038/s41598-024-56322-9 (PMC10937937; doi:10.1038/s41598-024-56322-9)
Supplement: Supplementary file 1 — Supplementary Table 1. [file 41598_2024_56322_MOESM1_ESM.docx]

| **Supplemental Table 1 -**  **Data relative to patients not included** | | | | | |
| --- | --- | --- | --- | --- | --- |
|  | **Patient n°15** | **Patient n°16** | **Patient n°17** | **Patient n°18** | **Patient n°19** |
| **Reason for non-inclusion** | No AKI | No AKI | No AKI | long-term treatment | long-term treatment |
| **Indication for CMX** | COPD exacerbation | Pneumocystis prophylaxis | Suspected pneumocystis | Nocardia | Pneumocystis prophylaxis |
| **Age** | 31 | 16 | 59 | 64 | 63 |
| **Gender** | M | M | M | M | F |
| **BMI (kg/m2)** | 24 | NA | 31,1 | 21.1 | 22.1 |
| **Arterial hypertension** | No | No | Yes | No | No |
| **Diabetes mellitus** | No | No | Yes | No | No |
| **Immunocompromised** | Yes | Yes | Yes | Yes | Yes |
| **Chronic kidney disease (stage)** | No | Yes (3A) | No | No | No |
| **Glomerular filtration rate (GFR) at the time of crystalluria (mL/min/1.73m2)** | 114 | 59 | 98 | 29 | 17 |
| **Serum creatinine at admission (µmol/L)** | 79 | 150 | 70 | 205 | 261 |
| **Serum albumine at admission (mmol/L)** | 34 | NA | NA | 32 | 28.7 |
| **Initial dose of SMX (mg/kg/day)** | 49,23 (PO) | 10,6 (PO) | 88,88 (IV) | 52,46 (PO) | 3.14 |
| **Cumulative SMX dose received prior crystalluria** | 25600 | NA | 23 600 | NA | NA |
| **AKI severity (KDIGO classification)** | No AKI | No AKI | No AKI | 2 | 3 |
| **Serum creatinine on the day of crystalluria (µmol/L)** | 56 | NA | 70 | 205 | 261 |
| **Use of other potentially nephrotoxic drugs** | No | No | Yes (Amikacin) | No | 17 |
| **Serum creatinine 3 to 6 months after crystalluria (µmol/L)** | 69 | NA | 82 | NA | NA |
| **Glomerular filtration rate (GFR) 3 to 6 months after crystalluria (mL/min/1.73m2) (CKD-EPI)** | 120 | NA | 90 | NA | NA |
| *AKI: Acute Kidney Injury ; COPD : Chronic Obstructive Pulmonary Disease ; CMX : Cotrimoxazole ; SMX : Sulfamethoxazole ; IV : Intravenously ; PO : per-os ; NA : data not available* | | | | | |
