# Supplementary material for: Sulfamethoxazole-induced crystal nephropathy: characterization and prognosis in a case series
Source: Sci Rep. 2024 Mar 13;14:6078. doi: 10.1038/s41598-024-56322-9 (PMC10937937; doi:10.1038/s41598-024-56322-9)
Supplement: Supplementary file 2 — Supplementary Table 2. [file 41598_2024_56322_MOESM2_ESM.docx]

| **Supplemental Table 2 - Naranjo Score in individuals** | | | | | | | |
| --- | --- | --- | --- | --- | --- | --- | --- |
| **Patient** | **n°1** | **n°2** | **n°3** | **n°4** | **n°5** | **n°6** | **n°7** |
| **Question 1** | 1 | 1 | 1 | 1 | 1 | 1 | 1 |
| **Question 2** | 2 | 2 | 2 | 2 | 2 | 2 | 2 |
| **Question 3** | 1 | 1 | 1 | 1 | 1 | 1 | 1 |
| **Question 4** | 0 | 0 | 0 | 0 | 0 | 0 | 0 |
| **Question 5** | 2 | 2 | -1 | 2 | 0 | 0 | 2 |
| **Question 6** | 0 | 0 | 0 | 0 | 0 | 0 | 0 |
| **Question 7** | 0 | 0 | 0 | 0 | 0 | 0 | 0 |
| **Question 8** | 1 | 0 | 1 | 1 | 0 | 0 | 0 |
| **Question 9** | 0 | 0 | 0 | 0 | 0 | 0 | 0 |
| **Question 10** | 1 | 1 | 1 | 1 | 1 | 1 | 1 |
| **Total score** | 8 | 7 | 5 | 5 | 5 | 5 | 7 |
| **Patient** | **n°8** | **n°9** | **n°10** | **n°11** | **n°12** | **n°13** | **n°14** |
| **Question 1** | 1 | 1 | 1 | 1 | 1 | 1 | 1 |
| **Question 2** | 2 | 2 | 2 | 2 | 2 | 2 | -1 |
| **Question 3** | 1 | 1 | 1 | 1 | 0 | 0 | 1 |
| **Question 4** | 0 | 0 | 0 | 0 | 0 | 0 | 0 |
| **Question 5** | 2 | -1 | 2 | 2 | -1 | -1 | -1 |
| **Question 6** | 0 | 0 | 0 | 0 | 0 | 0 | 0 |
| **Question 7** | 0 | 0 | 0 | 0 | 0 | 0 | 0 |
| **Question 8** | 0 | 1 | 0 | 0 | 1 | 0 | 0 |
| **Question 9** | 0 | 0 | 0 | 0 | 0 | 0 | 0 |
| **Question 10** | 1 | 1 | 1 | 1 | 1 | 1 | 1 |
| **Total score** | 7 | 6 | 7 | 7 | 4 | 3 | 1 |
| *Patients numbered 1 to 11 are those for whom the imputability of CMX to AKI according to the Naranjo score is probable (score ≥5).*  *Patients numbered 12 to 14 are those for whom imputability is possible (score ≤4).* | | | | | | | |
